# Evolution of the Gut Microbiome in Early Childhood: A Cross-Sectional Study of Chinese Children

**DOI:** 10.3389/fmicb.2020.00439

**Published:** 2020-04-03

**Authors:** Jing Niu, Long Xu, Yun Qian, Zhuo Sun, Dongbao Yu, Jiandong Huang, Xiaolin Zhou, Yizhong Wang, Ting Zhang, Rongrong Ren, Zhengpeng Li, Jialin Yu, Xuefeng Gao

**Affiliations:** ^1^Department of Pediatrics, Shenzhen University General Hospital, Shenzhen, China; ^2^Shenzhen University Clinical Medical Academy, Shenzhen, China; ^3^Department of Gastroenterology and Hepatology, Shenzhen University General Hospital, Shenzhen, China; ^4^Shenzhen Hoiracle Bio-Tech Co., Ltd., Shenzhen, China; ^5^Department of Gastroenterology, Hepatology, and Nutrition, Shanghai Children’s Hospital, Shanghai Jiao Tong University, Shanghai, China; ^6^Department of Gastroenterology and Hepatology, Chinese PLA General Hospital, Beijing, China

**Keywords:** gut microbiome, Chinese children, birth mode, gender, geographical location, gastrointestinal tract symptoms

## Abstract

Temporal development of the human gut microbiome from infancy to childhood is driven by a variety of factors. We surveyed the fecal microbiome of 729 Chinese children aged 0–36 months, aiming to identify the age-specific patterns of microbiota succession, and evaluate the impact of birth mode, gender, geographical location, and gastrointestinal tract symptoms on the shaping of the gut microbiome. We demonstrated that phylogenetic diversity of the gut microbiome increased gradually over time, which was accompanied by an increase in *Bacteroidetes* and a reduction in *Proteobacteria* species. Analysis of community-wide phenotypes revealed a succession from aerobic bacteria and anaerobic bacteria to facultative anaerobes, and from Gram-negative to Gram-positive species during gut microbiota development in early childhood. The metabolic functions of the gut microbiome shifted tremendously alongside early physiological development, including an increase in alanine, aspartate, and glutamate metabolism, and a reduction in glutathione, fatty acid, and tyrosine metabolism. During the first year of life, the *Bacteroidetes* phylum was less abundant in children born by casarean section compared with those delivered vaginally. The *Enterococcaceae* family, a group of facultative anaerobic microorganisms with pathogenic potential, was predominant in preterm infants. No measurable effect of maternal antibiotic exposure on gut microbiota development was found in the first 3 years of life. The relative abundances of *Coriobacteriaceae* and *Streptococcaceae* families, and *Megasphaera* genus were found to be higher in girls than in boys. Among the three first-tier Chinese cities, children born and fed in Beijing had a higher abundance of *Enterococcaceae* and *Lachnospiraceae* families, and Shenzhen children had a higher abundance of *Fusobacteriaceae*. The families *Alcaligenaceae*, *Bacteroidaceae*, and *Porphyromonadaceae* were more abundant in children with constipation, whereas the relative abundance of the *Clostridium* genus was higher in those with diarrhea.

## Introduction

The development of the human gut microbiome in early life has lasting effects on the host, and aberrancies in this process may affect health in adulthood (Rautava et al., 2012; Arrieta et al., 2014). Gut microbial alterations during early infancy have been shown to affect the risk of childhood obesity (Koleva et al., 2015), type 1 diabetes (Kostic et al., 2015), non-alcoholic fatty liver disease (NAFLD) (Nash et al., 2017), asthma (Arrieta et al., 2015), and allergic disease (Low et al., 2017; Candy et al., 2018). Therefore, an understanding of gut microbiota development in early life could provide insight into how its development impacts immune development, and lead to microbial-based therapeutics that target disease prevention at an early age. Gut microbiota development can be affected by a range of early life events, such as the mode of delivery (cesarean section versus vaginal delivery), maternal antibiotic usage, breast feeding/formula/mixed feeding, and the cession of breast-feeding (Penders et al., 2006; Gronlund et al., 2007; Dominguez-Bello et al., 2010; Ladirat et al., 2013; Azad et al., 2014; Nylund et al., 2014; Voreades et al., 2014). To date, relatively few studies have investigated the developmental patterns of the gut microbiome during early life in large, multicenter populations.

The Environmental Determinants of Diabetes in the Young (TEDDY) study is a collaborative effort between six research centers in the United States and Europe, aimed at uncovering the environmental causes of type I diabetes (Vatanen et al., 2018). The TEDDY study population produced a robust analysis of gut microbiome development in 783 infants from the age of 3 to 48 months. Using metagenomic sequencing, the TEDDY study demonstrated that the infant microbiome, both in terms of its composition and function, underwent dynamic changes in the first year of life that were exclusively dominated by three *Bifidobacterium* species (*B. bifidum, B. breve*, or *B. longum*) or by the Proteobacteria phylum. Individual variations were most significant during the early months, and inter-subject differences contributed to 35% of the microbial taxonomic variation (Vatanen et al., 2018). Moreover, factors including age, geographical location, and feeding type also had strong effects on the composition of the gut microbiome (Vatanen et al., 2018). Despite the dynamic and individualized nature of infant microbiome development, the TEDDY study identified a range of consistent patterns in the changes of microbial metabolic enzymes, which reflected major shifts in microbial structure and diet during infant microbiome development (Vatanen et al., 2018).

Given the importance of gut microbial development, a new perspective proposes to use microbes as clues to understand human postnatal development (Subramanian et al., 2015). However, the evolution of gut microbial development during early life remains poorly understood. Moreover, the infant microbiome in a predominantly Chinese population has never been explored (Bokulich et al., 2016). Here, we profiled the gut microbial development of a large cohort of Chinese infants during the first 36 months of life and evaluated the effect of delivery modes and digestive disorders on the composition of the gut microbial community.

## Materials and Methods

### Study Population

A total of 1156 children under 3 years of age were enrolled in this study during January 2016 and December 2018. Written informed consent was obtained from parents prior to study. The collection and use of infant fecal samples were approved by the Regional Ethical Review Board of the Shanghai Children’s Hospital. Infant stool samples were collected by parents, enclosed in 2-ml tubes containing 1.2 ml RNAlater (QIAGEN, Hilden, Germany), and delivered to the HRK (Hoiracle Bio-Tech Co., Ltd., Shenzhen, China.)-biotech laboratory within 48 h. All samples were stored at −80°C. Parents were asked to complete questionnaires and provide metadata regarding pregnancy and medical records of the mothers, as well as provide the delivery mode, feeding, and medical records of the infants. Children were classified as having constipation if they had <1 stool/day before the introduction of solid foods or <3 stools/week after the introduction of solid foods. Diarrhea was defined as watery/loose stools that increased in frequency compared to usual. Any subjects who had birth defects, genetic diseases, metabolic disorders (diabetes or cholestasis), or used systemic antibiotics (intravenous, intramuscular, or oral) one month before sample collection were excluded from this study.

### Fecal Microbiome Analysis

The QIAamp Fast DNA Stool Mini Kit (QIAGEN, Hilden, Germany) was used to isolate total microbial DNA from the stool samples using a previously reported procedure (Wang et al., 2018). The 16S rRNA V3-V4 hypervariable regions (encompassing approximately 469 bp) were PCR-amplified with dual-indexed PCR primers. The same procedures for DNA extraction and PCR amplification were applied to water samples as a control. The size and quality of the purified amplicons were checked with an Agilent 2100 Bioanalyzer (Agilent Technologies, Palo Alto, CA, United States). An equal amount of each amplicon was pooled and sequenced using the Illumina MiSeq platform, producing 300 or 250 bp paired-end reads.

Raw forward and reverse reads were joined and quality trimmed using USEARCH v10.0.240 with the -*fastq_filter* command and -*fastq_maxee* 1.0. After filtering, samples having less than 5000 reads were removed, resulting in 729 samples for later analyses. USEARCH’s *fastx_uniques* command was used to generate a FASTA file containing unique sequences. NOISE3 was performed to remove chimeras in the unique sequences and generate a BIOM table with representative zero-radius operational taxonomic units (OTUs) for subsequent analyses. The greengenes 13_5 reference database was employed for taxonomic assignment (DeSantis et al., 2006).

The OTU counts were normalized by total sum normalization (TSS) using Calypso (version 8.84) (Zakrzewski et al., 2017), and cumulative-sum scaling (CSS) was applied to correct biases introduced by TSS. Data were then log_2_ transformed to account for the non-normal distribution of taxonomic count data. Gut microbiome alpha diversity was measured using the Shannon, Chao1, and Simpson indices. The beta diversity between samples was calculated through Principal Coordinate Analysis (PCoA) using the Bray-Curtis distance based on genus abundance. Statistical differences in alpha diversity were determined by the Wilcoxon rank-sum test for the comparison of two groups, and one-way ANOVA followed by Tukey’s test for a comparison of three or more groups. An analysis of the composition of microbiomes (ANCOM) was performed on the top 300 most abundant taxa at the phylum and family levels to determine differentially abundant taxa (Mandal et al., 2015). ANCOM accounts for compositionality of microbiota survey data by reducing false discoveries in detecting differentially abundant taxa (Weiss et al., 2017).

KEGG Ortholog functional profiles of microbial communities were predicted using PICRUSt (Langille et al., 2013) based on the OTUs identified by QIIME 1.9 (Caporaso et al., 2010), and the statistical significance was determined by the Linear Discriminant Analysis (LDA) Effect Size (LEfSe) method (Segata et al., 2011). Microbiome phenotypes at the organism-level were predicted and analyzed using BugBase (Ward et al., 2017). The Spearman correlation coefficient was used to demonstrate the associations between the age of children and the gut microbiome composition, as well as the PICRUSt-predicted metabolic pathways.

## Results

### Study Cohort

As shown in Table 1, 729 children from 27 regions (provinces or cities) across China were selected for analysis, including 317 girls (43.5%) and 412 boys (56.5%), to investigate the evolutionary dynamics of the gut microbiome. The mean age of the children was 11 ± 8.6 months, and the mean gestational age was 39 ± 1.8 weeks. The characteristics of study participants from each region are given in Supplementary Table S1. We classified the children into twelve age groups: from birth to 3 months (*n* = 65), 4–6 months (*n* = 95), 7–9 months (*n* = 131), 10–12 months (*n* = 130), 13–15 months (*n* = 91), 16–18 months (*n* = 65), 20–21 months (*n* = 36), 22–24 months (*n* = 30), 25–27 months (*n* = 11), 28–30 months (*n* = 22), 31–33 months (*n* = 27), and 34–36 months (*n* = 26). 471 children (64.6%) were born via vaginal delivery, and 258 (35.4%) were born by cesarean section. 86 (11.8%) were constipated at the time of sample collection, and 75 (10.29%) had diarrhea.

**TABLE 1:** Characteristics of the study population.

Category	n (%)
Children age at the time of sample collection, month (IQR)	11 (7, 17)
Gestational age, weeks (IQR)	39 (38, 40)
Maternal age at the time of delivery, years (IQR)	30 (27, 33)
Maternal BMI (IQR)	26.9 (25.2, 28.7)
**Gender**	
Boy	412 (56.5%)
Girl	317 (43.5%)
**Birth mode**	
Vaginal delivery	471 (64.6%)
Cesarean section	258 (35.4%)
**Maternal antibiotics exposure**	
Maternal antibiotics treatment	51 (7.0%)
No maternal antibiotics exposure	678 (93.0%)
**Feeding type before 6 months of age**	
Breastfeeding	380 (52.13%)
Formula feeding	52 (7.13%)
Mixed feeding	297 (40.74%)
**Gastrointestinal symptoms**	
Constipation	86 (11.8%)
Diarrhea	75 (10.3%)
No GI symptom	568 (77.9%)

### Dynamics of Gut Microbiome Assembly During the First Three Years of Life

**FIGURE 1:**
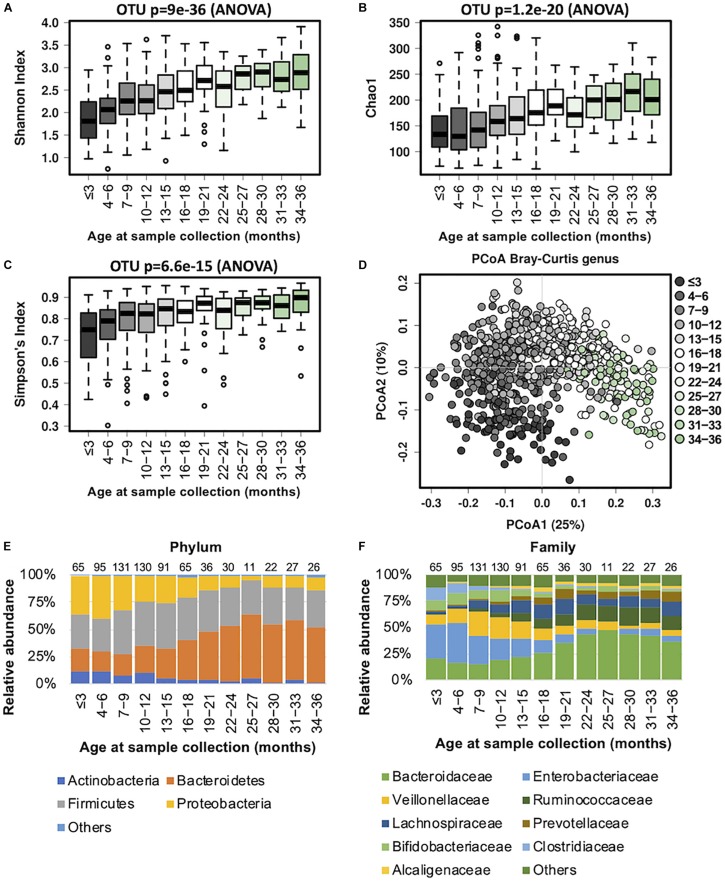
Fecal microbiota diversity and composition for 729 children according to age (0–3 years). Longitudinal alpha diversity from 0 to 36 months of age was measured by **(A)** Shannon index, **(B)** Chao1, and **(C)** Simpson’s index. **(D)** PCoA of Bray–Curtis distance based on the genus level. Each sample is colored according to age. Evolution of the gut microbiome at the **(E)** phylum and **(F)** family levels alongside physiological development. Number of children per age group is provided above bars.

Our data revealed that the gut microbiome alpha diversity increased dramatically from birth to 36 months (Figures 1A–C). A transverse gradient from birth to 36 months was obtained (Figure 1D), indicating progressive changes in the microbial community structure. Comparison of the compositional features of the gut microbiota alongside the age spectrum revealed several characteristic patterns. At the phylum level, *Bacteroidetes*, *Firmicutes*, *Proteobacteria*, and *Actinobacteria* were the dominant bacteria across the first 3 years of life (Figure 1E). The abundance of *Bacteroidetes* increased dramatically from birth to 27 months, and then remained stable until 36 months, while the abundance of *Proteobacteria* showed an opposite trend, with a significant decrease from birth to 24 months. The relative abundance of *Firmicutes* remained relatively stable over time (Figure 1E). At the family level, the relative abundances of *Bacteroidaceae, Prevotellaceae*, and *Ruminococcaceae* increased progressively from birth to 36 months, and *Ruminococcaceae* showed a significant positive correlation with age (*R* = 0.61, Table 2). In contrast, *Enterobacteriaceae* and *Veillonellaceae* gradually decreased during the first 3 years of life (Figure 1F). *Enterobacteriaceae* and *Enterococcaceae* were negatively correlated with age, with *R* = −0.59 and −0.53, respectively (Table 2).

**TABLE 2:** Taxa in the gut microbiome that significantly correlate with age.

Taxa	Spearman |R| > 0.45	*P*-value	Abundance (Mean, SE)	Positive samples (n,%)
**Family**				
*Ruminococcaceae*	0.61	4.58E-76	(6.12%, 0.35%)	(722, 99.04%)
*Enterobacteriaceae*	–0.59	9.96E-69	(20.9%, 0.76%)	(729, 100%)
*Enterococcaceae*	–0.53	1.78E-54	(0.37%, 0.12%)	(549, 75.31%)
**Genus**				
*Blautia*	0.58	3.69E-67	(0.82%, 0.06%)	(643,88.2%)
*Coprococcus*	0.56	1.40E-61	(0.21%, 0.02%)	(570, 78.19%)
*Faecalibacterium*	0.51	1.84E-50	(4.36%, 0.31%)	(680, 93.28%)
*Enterococcus*	–0.55	9.86E-58	(0.37%, 0.12%)	(549, 75.31%)
*Erwinia*	–0.52	4.63E-52	(0.65%, 0.07)	(682, 93.56%)
*Dorea*	0.49	4.01E-45	(0.78%, 0.08%)	(641, 87.93%)

Progressive changes in microbial diversity and abundance of the infant gut microbiome are likely to re-shape the metabolic functions of the host over time. We used BugBase to infer common microbial phenotypes from our 16S rRNA sequencing data, including the anticipated oxygen tolerance and Gram staining of present species. Along the lines of previous reports (Backhed et al., 2015; Bokulich et al., 2016; Chu et al., 2017; Nagpal et al., 2017), our data showed that the early gastrointestinal tract is predominantly populated with facultative anaerobes (such as *Enterococcaceae*), and progressively shifts to obligate anaerobic microorganisms, resulting in a decreased oxidative stress tolerance (Supplementary Figure S1). The ratio of Gram-negative to Gram-positive bacterial species was higher at the beginning of life, and significantly decreased alongside physical growth. The proportion of mobile element containing, biofilm forming, and pathogenic bacteria also decreased with age.

We also observed that the predicted metabolic functions went through dynamic changes during the first 3 years of child development (Supplementary Figure S2A). The KEGG pathways associated with transcription machinery, streptomycin biosynthesis, oxidative phosphorylation, methane metabolism, alanine, aspartate and glutamate metabolism, drug metabolism other enzymes, and one carbon pool by folate were progressively upregulated over time (with Spearman correlation |R| > 0.45 and *p* < 0.05; Table 3). In contrast, glutathione metabolism, inorganic ion transport and metabolism, valine, leucine and isoleucine degradation, ubiquinone and other terpenoid quinone, fatty acid metabolism, the phosphotransferase system, the secretion system, propanoate metabolism, the bacterial secretion system, and tyrosine metabolism were downregulated over time. Together, these changes indicate a clear trajectory of gut microbiome development in the first 3 years of life.

**TABLE 3:** Predicted functions of the gut microbiome that significantly correlate with age.

KEGG pathways	Spearman |R| > 0.45	*P*-value	Abundance (Mean, SE)	Positive samples (n,%)
Transcription machinery	0.59	1.30E-69	(0.84%, 0.91%)	(729,100%)
Streptomycin biosynthesis	0.48	1.84E-43	(0.30%, 0.34%)	(729,100%)
Oxidative phosphorylation	0.48	2.66E-43	(1.06%, 0.55%)	(729,100%)
Methane metabolism	0.48	3.38E-43	(1.08%, 0.46%)	(729,100%)
Alanine, aspartate and glutamate metabolism	0.47	6.61E-42	(1.03%, 0.68%)	(729,100%)
Drug metabolism other enzymes	0.46	5.01E-40	(0.29%, 0.17%)	(729,100%)
One carbon pool by folate	0.46	8.52E-39	(0.56%, 0.39%)	(729,100%)
Tyrosine metabolism	–0.46	9.11E-40	(0.36%, 0.18%)	(729,100%)
Bacterial secretion system	–0.46	2.71E-39	(0.72%, 0.59%)	(729,100%)
Propanoate metabolism	–0.47	3.65E-41	(0.54%, 0.24%)	(729,100%)
Secretion system	–0.51	2.06E-50	(1.45%, 1.74%)	(729,100%)
Phosphotransferase system (PTS)	–0.52	1.56E-52	(0.65%, 1.37%)	(729,100%)
Fatty acid metabolism	–0.53	3.28E-54	(0.29%, 0.32%)	(729,100%)
Ubiquinone and other terpenoid quinone biosynthesis	–0.53	2.53E-53	(0.32%, 0.35%)	(729,100%)
Valine, leucine and isoleucine degradation	–0.55	1.09E-57	(0.28%, 0.25%)	(729,100%)
Inorganic ion transport and metabolism	–0.6	3.75E-73	(0.31%, 0.36%)	(729,100%)
Glutathione metabolism	–0.6	4.76E-72	(0.29%, 0.25%)	(729,100%)

### Birth Mode Related Differences in the Gut Microbiome of Young Children

**FIGURE 2:**
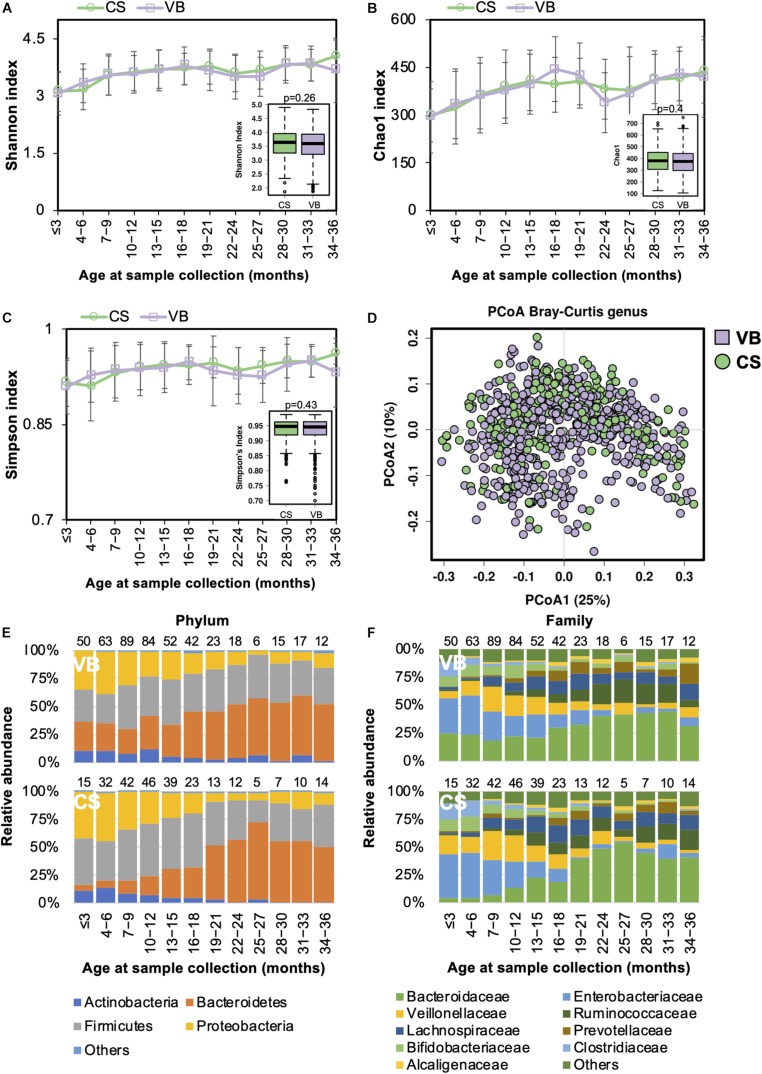
Fecal microbiota diversity and composition for 729 children according to delivery mode. Alpha diversity, which was calculated by **(A)** Shannon index, **(B)** Chao1, or **(C)** Simpson’s index, showed no significant difference between the two birth modes. **(D)** PCoA showed no clear separation between samples based on the two birth modes. Birth-mode-stratified taxa summary composition of bacterial microbiota at the **(E)** phylum and **(F)** family levels. Number of children per age group is provided above bars.

Casarean section (C-section) accounts for 35% of all deliveries in China (35.4% from our study cohort), which is higher than in many other developed countries (Li et al., 2017). Birth mode was shown to be significantly associated with the gut microbiome during the developmental phase (Stewart et al., 2018). We compared the gut microbiome between the two birth modes and found no significant difference in the alpha diversity (Figures 2A–C). PCoA analysis of the beta diversity further indicated that the microbial composition of both modes could be superimposed onto each other (Figure 2D). During the first 18 months of life, the Firmicutes and Proteobacteria phyla predominated in children born via C-section, whereas Bacteroidetes were more abundant in those delivered by vaginal birth (Figure 2E). At the family level, the abundance of *Enterococcaceae* was higher in C-section-delivered children during the first year of life, whereas *Bacteroidaceae* was enriched in those born vaginally (Figure 2F). The difference in the gut microbiome composition between the two birth modes diminished after the age of 12 months. Overall, the relative abundance of the *Bacteroidaceae* family and *Bacteroides* genus were significantly higher in vaginally born infants compared with those who were born via C-section (ANCOM *p* < 0.05, Supplementary Table S2).

Functionally, the relative abundance of transporter genes was more abundant in children delivered via C-section (Supplementary Figure S2B).

### Gut Microbiome Development Is Similar Between Preterm and Term Children

**FIGURE 3:**
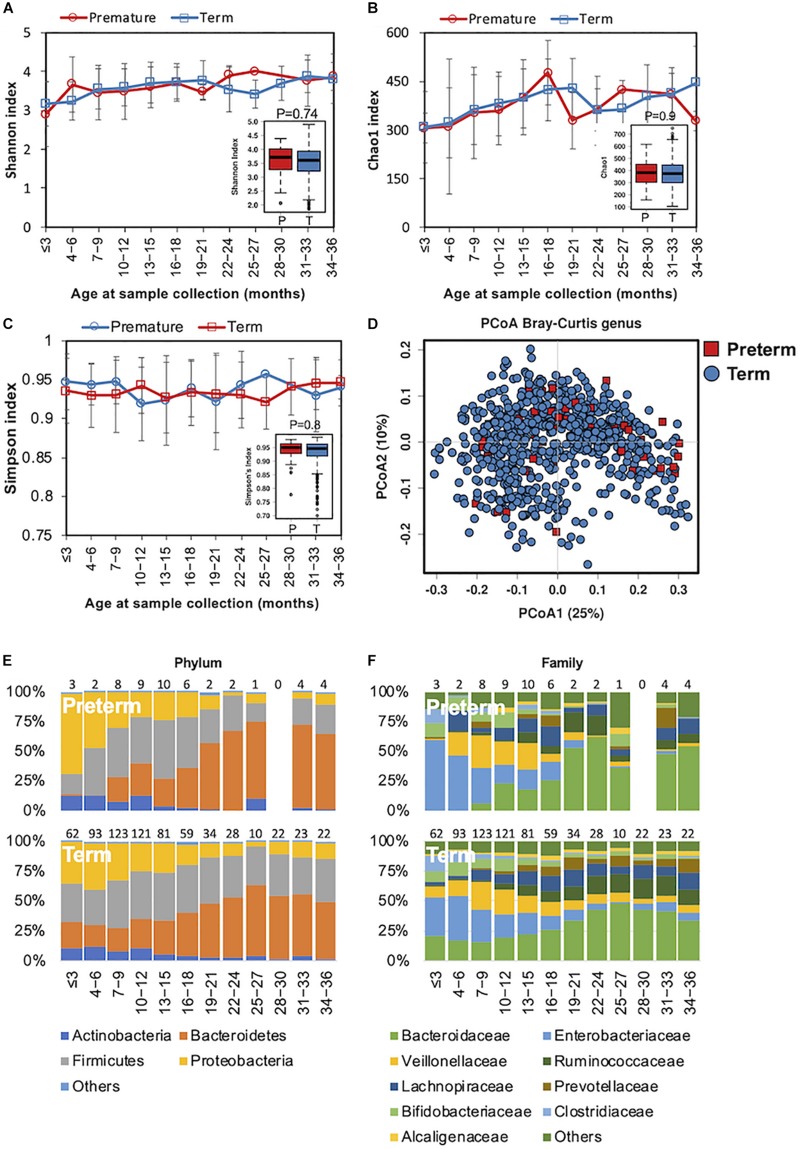
Fecal microbiota diversity and composition for 729 children according to gestational age. Alpha diversity was calculated with **(A)** Shannon index, **(B)** Chao1, and **(C)** Simpson’s index, and showed no significant difference between premature and full-term children. **(D)** PCoA showed no clear separation between samples from premature and full-term children. Gestational age-stratified taxa summary composition of bacterial microbiota at the **(E)** phylum and **(F)** family levels. Number of children per age group is provided above bars.

In preterm infants, microbial colonization is challenging due to organ immaturity, the length of hospital stay, and the use of antibiotics. In our cohort, 51 children were preterm, born with gestational ages less than 37 weeks (Table 1). The complexity of the microbiota increased in both preterm children and in those children born at full term simultaneously (Figures 3A–C). Beta diversity analysis with PCoA revealed that the gut bacterial community structure was not distinguishable between preterm and term children (Figure 3D). During the first 6 months, *Enterococcaceae* (Proteobacteria phylum), a group of facultative anaerobic microorganisms with pathogenic potential, was predominant in preterm infants (Figures 3E,F). This was in line with previous studies showing that the gut microbiota of preterm infants was impacted by a bloom of opportunistic and potentially pathogenic bacteria (Arboleya et al., 2012; Barrett et al., 2013). Children born preterm showed a lower relative abundance of *Blautia* genus than those born full term (ANCOM *p* < 0.05, Supplementary Table S3). No significant difference in the gut microbiota functional profile was detected between children born preterm and those born full term.

### Maternal Antibiotic Exposure During Pregnancy May Not Affect the Early Gut Microbiota

**FIGURE 4:**
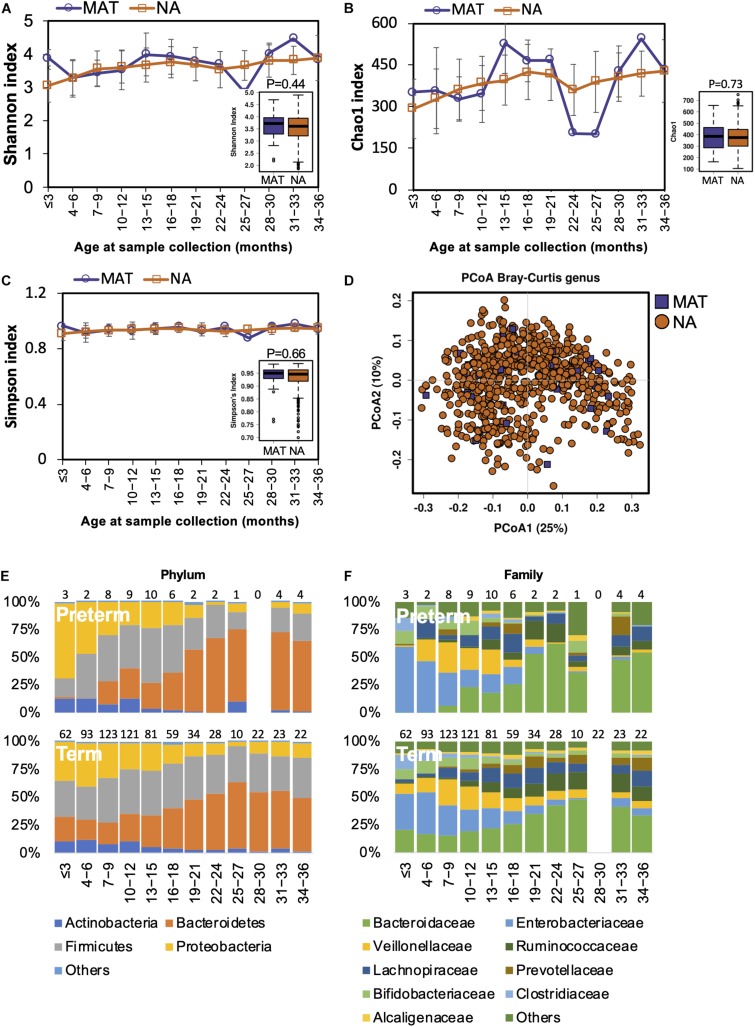
Fecal microbiota diversity and composition for 729 children according to maternal antibiotic exposure. Alpha diversity, which was calculated with **(A)** Shannon index, **(B)** Chao1, and **(C)** Simpson’s index, showed no significant difference between MAT and NA children. **(D)** PCoA showed no clear separation between samples from MAT and NA children. The gut microbiota development patterns in MAT and NA children at the **(E)** phylum and **(F)** family levels. Number of children per age group is provided above bars.

Both animal and human studies have shown that antibiotic use by mothers during pregnancy confers an altered microbiota to their offspring (Mueller et al., 2015; Gonzalez-Perez et al., 2016; Kuperman and Koren, 2016). In our study, the mothers of 40 children received antibiotic treatments during pregnancy. We examined if gut microbiota development was influenced by maternal antibiotics treatment (MAT). Alpha diversity in MAT children was not significantly different to those whose mothers had no antibiotic exposure (NA) during pregnancy (Figures 4A–C). The compositional structure of the gut microbiota could not be distinguished by beta diversity analysis with PCoA (Figure 4D). The pattern of gut microbiota development was not disturbed by maternal antibiotic exposure at the phylum or family levels (Figures 4E,F). During the first 3 years of life, there were no significant differences in the gut microbiota composition (at the phylum, family, or genus levels) or functions between children based on maternal antibiotic exposure.

### Gut Microbiome Development in Different Genders

**FIGURE 5:**
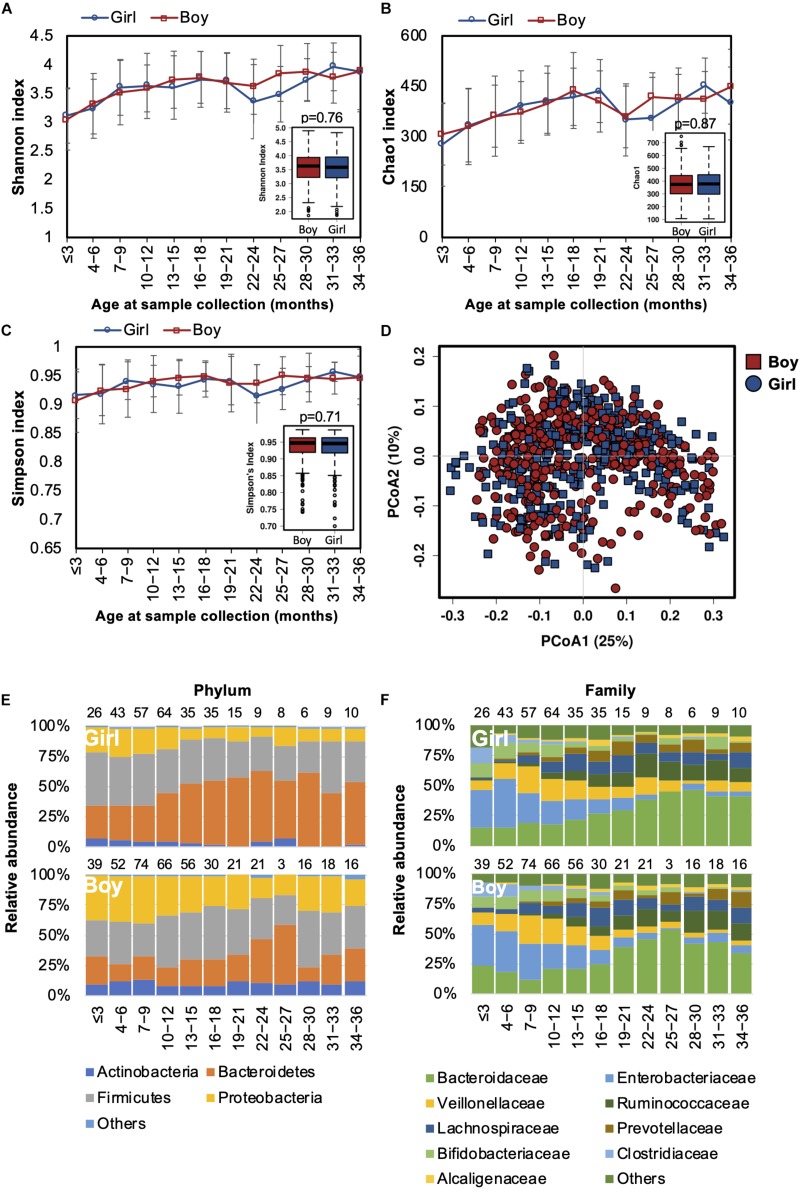
Fecal microbiota diversity and composition for 729 children based on gender. Alpha diversity was calculated with **(A)** Shannon index, **(B)** Chao1, or **(C)** Simpson’s index, and showed no significant difference between genders. **(D)** PCoA showed no clear separation of the gut microbiota composition between boys and girls. Gender-stratified taxa summary composition of bacterial microbiota at the **(E)** phylum and **(F)** family levels. Number of children per age group is provided above bars.

Differences in the gut microbiota of Chinese men and women were reported in our previous study (Gao et al., 2018). In this study, we also tested if the gender-associated differences in the gut microbiota originated from early life. Both alpha and beta diversity were not significantly different between genders (Figures 5A–D). The developmental trajectory of the gut microbiome was similar between genders (Figures 5E,F). Nevertheless, the relative abundances of *Coriobacteriaceae* and *Streptococcaceae* families and the *Megasphaera* genus were more abundant in girls (Supplementary Table S4). Based on the predicted function of metagenomes, no significant difference in the predicted metabolic functions were detected between genders.

### Geographical Location Affects the Gut Microbiome Composition of Young Children

**FIGURE 6:**
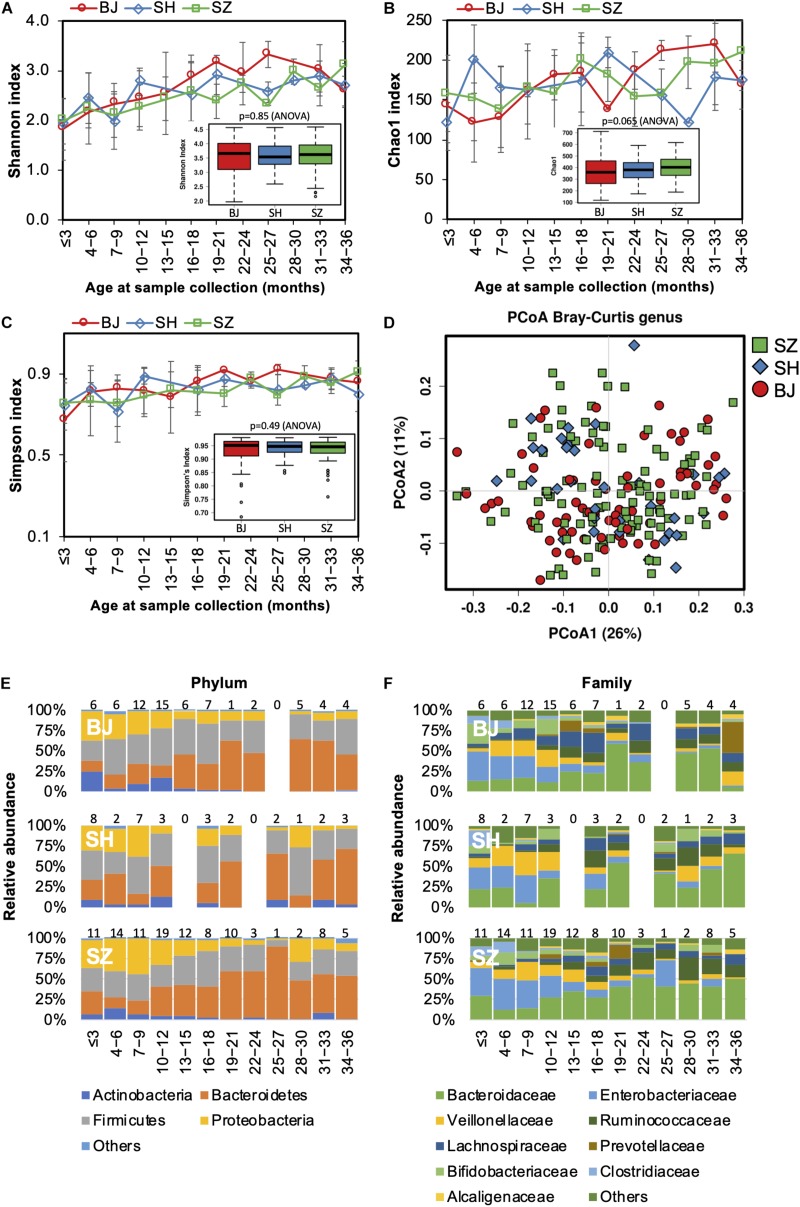
Fecal microbiota diversity and composition for 729 children according to geographical location. Alpha diversity, which was calculated with **(A)** Shannon index, **(B)** Chao1, and **(C)** Simpson’s index, showed no significant difference among the children from three first-tier cities of China. **(D)** PCoA showed no clear separation among samples of different geographical locations. Gut bacterial microbiota composition at the **(E)** phylum and **(F)** family levels. Number of children per age group is provided above bars.

As lifestyle and dietary patterns play an important role in shaping our gut microbiota, we compared gut microbiota biodiversity and composition of children born and raised in three first-tier cities in China from other distinct geographical areas, including Beijing, Shanghai, and Shenzhen. However, no significant difference in either alpha or beta diversity was detected between geographical groups (Figures 6A–D). The trajectory of gut microbiota development was similar among children from different cities (Figures 6E,F). Nevertheless, significant differences in the relative abundances of the gut microbiome were identified in the children from the three first-tier cities. A higher relative abundance of *Lachnospiraceae* family was observed in children from Beijing (ANCOM *p* < 0.05, Supplementary Table S5); the *Fusobacteriaceae* family (*Fusobacterium* genus) was more abundant in children from Shenzhen, while the relative abundance of *Enterococcaceae* was lower in children from Shanghai.

Geographical variation in the functional profile of the gut microbiome was not detected in our study.

### Alterations in the Gut Microbiome Are Associated With Gastrointestinal Disorders in Young Children

**FIGURE 7:**
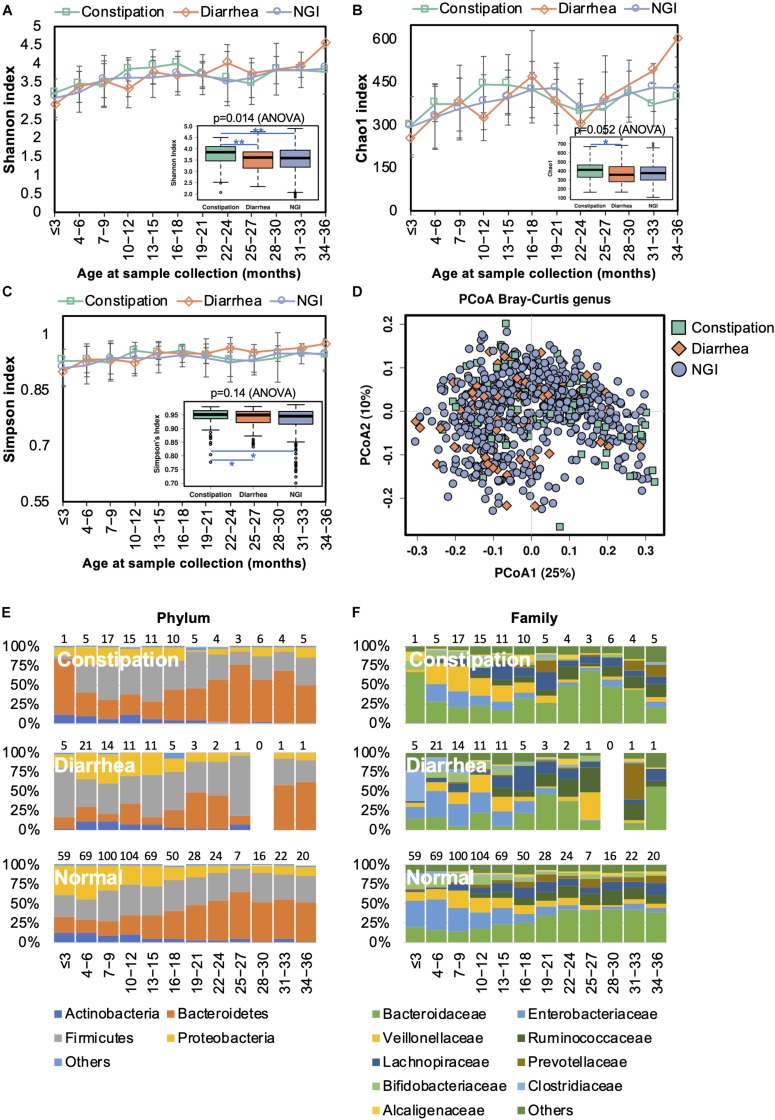
Fecal microbiota diversity and composition for 729 children according to gastrointestinal symptoms. Alpha diversity, which was calculated with **(A)** Shannon index, **(B)** Chao1, and **(C)** Simpson’s index, was found to be higher in children with constipation. **(D)** PCoA showed no clear separation among samples from children with and without GI symptoms. Gut bacterial microbiota composition at the **(E)** phylum and **(F)** family levels. Number of children per age group is provided above bars.

Changes in the gut microbiome have been shown to be associated with an increased incidence of gastrointestinal symptoms in early life (Mayer et al., 2014). We next characterized the gut microbiota in infants with constipation (<1 stool/day before the introduction of solid food or <3 stools/week after the introduction of solid food) or diarrhea (watery/loose stools that increase in frequency relative to usual). The bacterial alpha diversity was significantly higher in children with constipation than the alpha diversity in those with diarrhea and without GI symptoms (Figures 7A–C). PCoA results showed no clear separation between samples from children with and without GI symptoms (Figure 7D). Thus, GI disorders did not disturb the overall gut microbiota trajectory (Figures 7E,F). At the family level, however, *Alcaligenaceae*, *Bacteroidaceae*, and *Porphyromonadaceae* were significantly enriched in constipated children, while *Enterobacteriaceae* and *Gemellaceae* were diminished in them when compared with those with diarrhea or without GI symptoms (Supplementary Table S6). At the genus level, lower abundances of *Gemella* and *Salmonella*, and higher abundances of *Bacteroides* and *Blautia*, were observed in children with constipation compared to children without. There was a lower abundance of *Bacteroides* and a higher abundance of *Clostridium* in children with diarrhea compared to those with constipation or without GI symptoms.

Functional divergence associated with GI symptoms was detected by PICRUSt analysis. Functional genes associated with transporters, ABC transporters, the secretion system, transcription factors, the phosphotransferase system, the two component system, and bacterial motility proteins were significantly upregulated in the gut microbiome of children with diarrhea (Supplementary Figure S2C), whereas ribosome-related genes were enriched in the gut microbiome of children with constipation.

## Discussion

The initial colonization of the human gut microbiome is influenced by a wide range of factors that may have long-term physiological consequences (Dominguez-Bello et al., 2010). Consistent with previous studies (Koenig et al., 2011; Yatsunenko et al., 2012), our data shows that the infant gut microbiome undergoes dynamic changes during the first 36 months of life. Newborns have a low microbial diversity, which gradually increases thereafter. The structure of the microbiome community also undergoes dynamic changes during the first 36 months of life. Our data demonstrate that the infant gut microbiome was predominated by facultative anaerobes such as *Enterobacteriaceae* in the first months of life, which is in line with findings among infants from Japan (Nagpal et al., 2017), United States (Bokulich et al., 2016; Chu et al., 2017), and Europe (Backhed et al., 2015). This is because the newborn gut is a predominantly aerobic environment at birth (Arrieta et al., 2014). Then the gut gradually becomes anaerobic, thereby allowing the colonization of strict anaerobes such as *Bifidobacterium* (Actinobacteria phylum), *Clostridium* (Firmicutes phylum), and *Bacteroides* (Bacteroidetes phylum) (Matamoros et al., 2013). Consistently, we also found that fecal samples collected from infants aged 0–3 years were predominated by the phyla Proteobacteria and Firmicutes, followed by Bacteroidetes and Actinobacteria.

Despite similar developmental patterns in accordance with the timing of food change, the gut microbiota of infants from different countries exhibit different types of microbial composition. These are typified by P-type (abundant in Proteobacteria), A-type (abundant in Actinobacteria), and F-type (abundant in Firmicutes) developmental patterns (Kuang et al., 2016). Most infants from western countries are A-type, with 82% of American, 79% of Canadian, and 54% of Swedish infants being A-type, whereas most of those in Bangladeshi (70%) are F-type (Kuang et al., 2016). It has been reported that Chinese infants (age < 3 months) are predominately P-type (Kuang et al., 2016). Consistently, our data also demonstrated that Chinese infants aged 0–3 months were dominated by Proteobacteria. The establishment of a complex microbiota proceeds faster in developing rather than developed countries due to poor sanitation. For example, *Enterobacteriaceae* (Gram-negative bacteria) was found to be colonized in Chinese and Pakistani infants during the first weeks of life (Adlerberth et al., 1998), whereas this group of bacteria gradually increased their colonization rate in Swedish infants over the first 6 months of life (Adlerberth et al., 2006).

Although host genetic background accounts for a substantial fraction of the abundance of most common microbiota, the different microbial compositions observed in different studies can readily be explained by that fact that vaginal and skin microbial compositions may vary between mothers from different geographical locations (Huang et al., 2014; Gupta et al., 2017). It was reported that geographic location contributes more to the interindividual differences in the composition of the gut microbiome than other factors (He et al., 2018). In our data, differences in gut microbiota composition have also been observed within China. Among the enrolled subjects, children born and fed in Beijing had higher abundances of families *Enterococcaceae* and *Lachnospiraceae*, and the *Fusobacterium* genus was enriched in children from Shenzhen. Thus, regional variations should be considered when investigating the associations between gut microbiota and infant health and disease.

Diet plays a major role in the shifting of gut microbiota in early life. During the first few months of life, the primary source of nutrition is breast milk or formula. Therefore, the infant intestine at this stage favors the propagation of microbes such as *Bifidobacterium* that can ferment milk oligosaccharides. Introduction of solid food is a major contributor in the shift of the gut microbiome toward an adult-like structure, which was previously characterized by an increase of Bacteroidetes and the Firmicutes and a decrease in Actinobacteria and Proteobacteria phyla (Arrieta et al., 2014). The rationale behind this change is that the infant diet at this stage contains many polysaccharides that cannot be digested by their immature digestive system, thereby triggering an increase of the aforementioned microbes to facilitate this process. During 12–36 months of life, the infant gut microbiota develops progressively into an adult-like gut microbiota, which is dominated by Firmicutes and Bacteroidetes phyla (these make up >90% of the gut microbial population) (Ringel-Kulka et al., 2013; Voreades et al., 2014).

Previous studies have suggested that early patterns of the infant gut microbiota are impacted by delivery mode (Dominguez-Bello et al., 2010; Lozupone et al., 2013; Matamoros et al., 2013; Arrieta et al., 2014; Jakobsson et al., 2014). A relatively lower gut microbial diversity was previously reported in C-section-delivered infants at 2 years of age (Jakobsson et al., 2014). This decline in biodiversity has been attributed to delayed colonization of Bacteroidetes. Nevertheless, no difference in alpha diversity between the two birth modes was detected in our data. The microbial populations in the infant gut are generally considered to be similar to the microbiota the infant is exposed to at birth (Dominguez-Bello et al., 2010; Lozupone et al., 2013). For infants born by vaginal delivery, the intestinal microbiota resembles their mothers’ vaginal microbiota, which is dominated by *Lactobacillus* (phylum Firmicutes), *Prevotella* (phylum Bacteroidetes), or *Sneathia* (phylum Fusobacteria). In contrast, the gut microbiome of C-section-delivered infants is similar to the mothers’ skin microbiome, which has predominately *Staphylococcus* (phylum Firmicutes), *Corynebacterium* (phylum Actinobacteria), and *Propionibacterium* (phylum Actinobacteria) (Dominguez-Bello et al., 2010). A previous study of Chinese newborns (neonates and 2-month-olds) showed that vaginal delivery results in an enrichment of *Bacteroides*, *Parabacteroides*, and *Megamonas*, whereas C-section delivery led to an enrichment of *Prevotella*, *Streptococcus*, and *Trabulsiella* (Kuang et al., 2016). It is worth noting that *Prevotella*, which are enriched in vaginally delivered Amerindian infants (Dominguez-Bello et al., 2010), are also found in C-section-delivered Chinese infants (Kuang et al., 2016). Our data also shows that *Bacteroidaceae* was enriched in the gut of vaginally delivered infants, which was consistent with previous findings (Kuang et al., 2016). Taken together, these results suggest a role for the delivery mode of an infant in structuring the gut microbiota in early life.

Changes in gut microbiota composition have been associated with a variety of GI disorders, such as constipation and diarrhea. Children with functional constipation were shown with a higher fecal level of *Clostridium* and *Bifidobacterium* species (Zoppi et al., 1998). In adults with constipation, a depletion of members belonging to the *Bacteroides*, *Roseburia*, and *Coprococcus* phyla in the gut microbiota was observed (Mancabelli et al., 2017). Our data showed a variety of differences in the gut microbiome of children with constipation compared with their counterparts without any GI symptoms, as represented by an increased biodiversity and an increase in the relative abundance of families *Alcaligenaceae*, *Bacteroidaceae*, and *Porphyromonadaceae*. Regarding children with diarrhea, the *Clostridium* genus, including the pathogenic species, was found to be enriched. All together, these results show the close relationship between gut microbiota and gastrointestinal disorders in children.

## Conclusion

With a total of 729 children participating, this is the first comprehensive study of gut microbiome development in young Chinese children. From our results, the evolution of the gut microbiome during early life appears to be defined by shared phylogenetic trajectories. The infant gut microbiome is gradually established during the first 3 years of life. Factors such as birth mode, gestational age, antibiotic exposure, gender, geographical location and GI disorders could affect relative abundances of several groups of gut microbes.

## Data Availability Statement

The 16S rRNA sequence data reported in this study have been deposited in the European Nucleotide Archive (ENA) database, under accession number PRJEB35531.

## Ethics Statement

The studies involving human participants were reviewed and approved by The Regional Ethical Review Board in Shanghai Children’s Hospital. Written informed consent to participate in this study was provided by the participants’ legal guardian/next of kin.

## Author Contributions

XG and JY conceived the presented study. JN, ZS, RR, ZL, and YW collected the data. JH and XZ performed the sequencing. XG and DY performed the data analysis. JN and LX wrote the manuscript. YQ, YW, and TZ provided critical feedback and helped shape the research, analysis and manuscript.

## Conflict of Interest

DY, JH, and XZ were employed by Hoiracle Bio-Tech Co., Ltd. The remaining authors declare that the research was conducted in the absence of any commercial or financial relationships that could be construed as a potential conflict of interest.

## Supplementary Material

TABLE S1 Characteristics of the enrolled children from each region.

TABLE S2 Significant differences in the gut microbiota composition between two birth modes as determined using ANCOM.

TABLE S3 Significant differences in the gut microbiota composition between premature and full-term children as determined using ANCOM.

TABLE S4 Significant differences in the gut microbiota composition between genders as determined by ANCOM.

TABLE S5 Significant differences in the gut microbiota composition between children from three first-tier cities in China as determined using ANCOM.

TABLE S6 Significant differences in the gut microbiota composition between children with and without GI symptoms as determined using ANCOM.

FIGURE S1 Biological phenotypes of gut microbiome change over development. BugBase was employed to predict the proportion of aerobic, anaerobic, facultatively anaerobic, oxidative stress tolerant, Gram-negative, Gram-positive, mobile element containing, biofilm forming, and pathogenic bacteria within the gut microbiome of each age group. Kruskal–Wallis test was implemented to determine significant differences between age groups.

FIGURE S2 Prediction of metagenome functional content using PICRUSt. **(A)** The top 40 most abundant metabolism pathways evolved during the first 3 years of age. Metabolism pathways significantly different between children **(B)** born by vaginal delivery or C-section (CS), and **(C)** those with or without GI symptoms. The significance was determined by LEFSe, with an LDA score >3.0 and *p* < 0.05.
