# Supplementary figures and images for: Evolution of the Gut Microbiome in Early Childhood: A Cross-Sectional Study of Chinese Children
Source: Front Microbiol. 2020 Apr 3;11:439. doi: 10.3389/fmicb.2020.00439 (PMC7169428; doi:10.3389/fmicb.2020.00439)

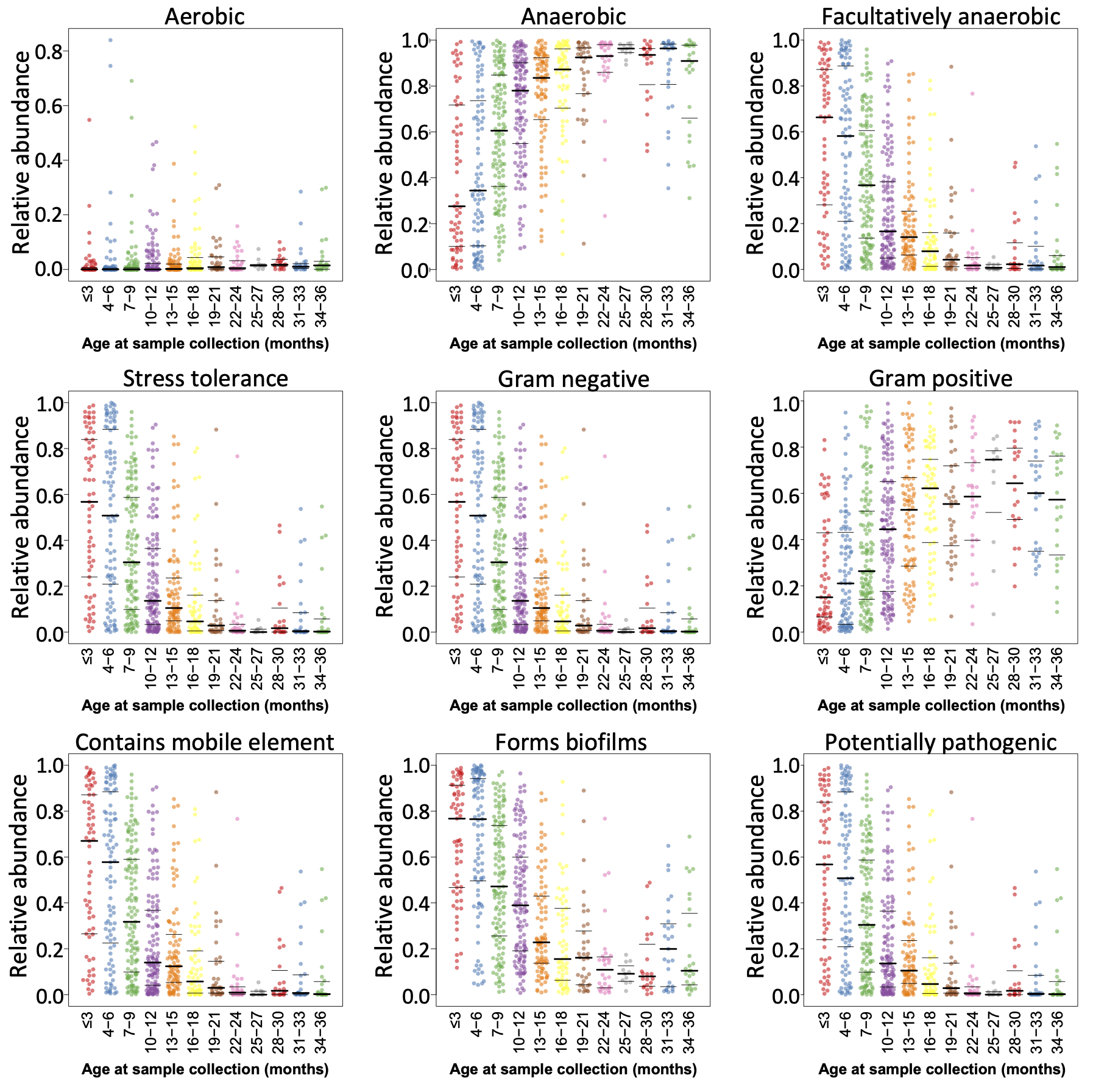

Supplement: FIGURE S1 — Biological phenotypes of gut microbiome change over development. BugBase was employed to predict the proportion of aerobic, anaerobic, facultatively anaerobic, oxidative stress tolerant, Gram-negative, Gram-positive, mobile element containing, biofilm forming, and pathogenic bacteria within the gut microbiome of each age group. Kruskal–Wallis test was implemented to determine significant differences between age groups. [file Image_1.TIFF]

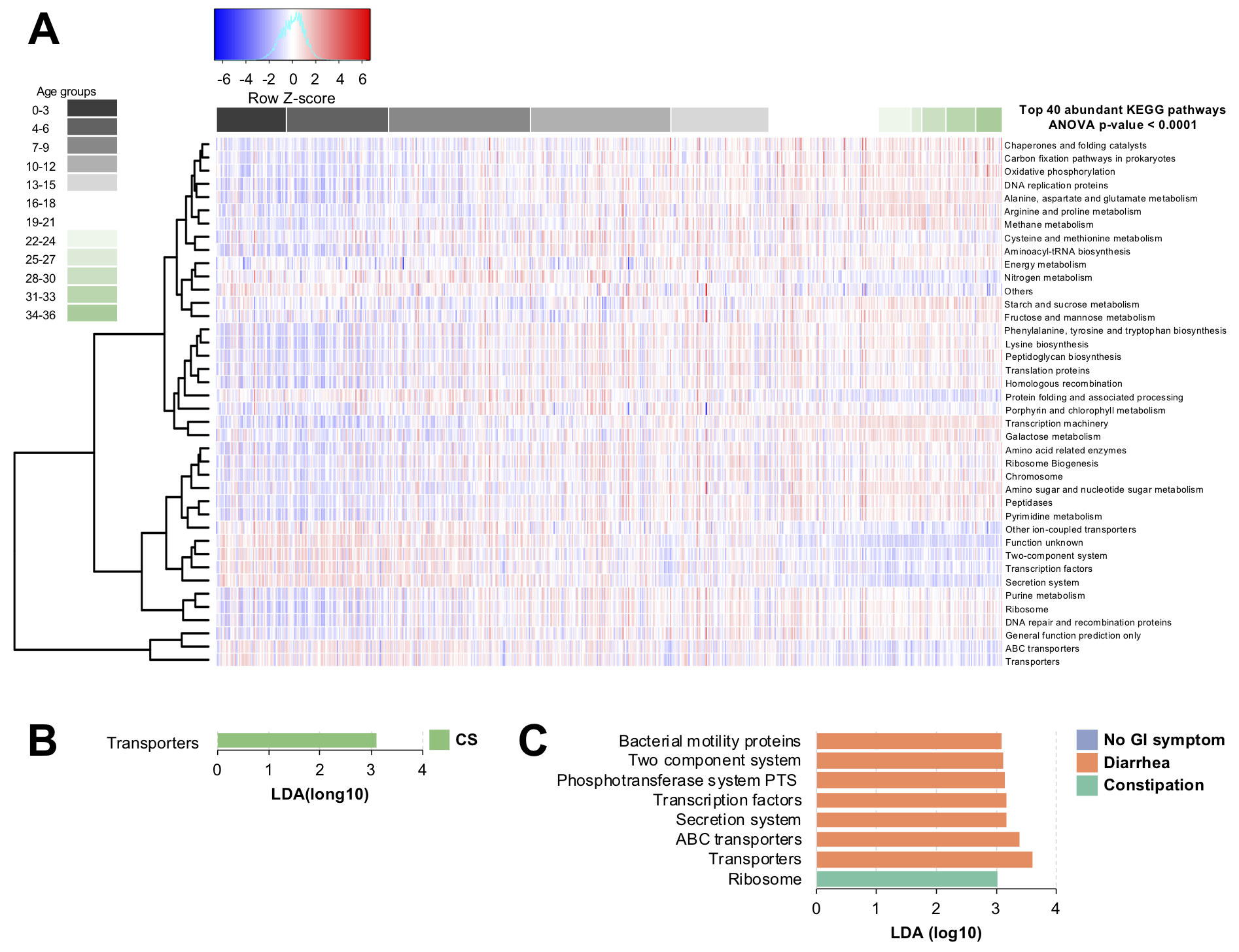

Supplement: FIGURE S2 — Prediction of metagenome functional content using PICRUSt. (A) The top 40 most abundant metabolism pathways evolved during the first 3 years of age. Metabolism pathways significantly different between children (B) born by vaginal delivery or C-section (CS), and (C) those with or without GI symptoms. The significance was determined by LEFSe, with an LDA score >3.0 and p < 0.05. [file Image_2.TIFF]
